# Molecular characterization of a rice mutator-phenotype derived from an incompatible cross-pollination reveals transgenerational mobilization of multiple transposable elements and extensive epigenetic instability

**DOI:** 10.1186/1471-2229-9-63

**Published:** 2009-05-29

**Authors:** Hongyan Wang, Yang Chai, Xiucheng Chu, Yunyang Zhao, Ying Wu, Jihong Zhao, Frédéric Ngezahayo, Chunming Xu, Bao Liu

**Affiliations:** 1 Key Laboratory of Molecular Epigenetics of MOE and Institute of Genetics & Cytology, Northeast Normal University, Changchun 130024, PR China; 2 Tonghua Academy of Agricultural Sciences, Hailong 135007, Jilin Province, PR China; 3 Key Laboratory of Applied Statistics of MOE, Northeast Normal University, Changchun 130024, PR China

## Abstract

**Background:**

Inter-specific hybridization occurs frequently in plants, which may induce genetic and epigenetic instabilities in the resultant hybrids, allopolyploids and introgressants. It remains unclear however whether pollination by alien pollens of an incompatible species may impose a "biological stress" even in the absence of genome-merger or genetic introgression, whereby genetic and/or epigenetic instability of the maternal recipient genome might be provoked.

**Results:**

We report here the identification of a rice mutator-phenotype from a set of rice plants derived from a crossing experiment involving two remote and apparently incompatible species, *Oryza sativa *L. and *Oenothera biennis *L. The mutator-phenotype (named Tong211-LP) showed distinct alteration in several traits, with the most striking being substantially enlarged panicles. Expectably, gel-blotting by total genomic DNA of the pollen-donor showed no evidence for introgression. Characterization of Tong211-LP (S0) and its selfed progenies (S1) ruled out contamination (via seed or pollen) or polyploidy as a cause for its dramatic phenotypic changes, but revealed transgenerational mobilization of several previously characterized transposable elements (TEs), including a MITE (*mPing*), and three LTR retrotransposons (*Osr7, Osr23 *and *Tos17*). AFLP and MSAP fingerprinting revealed extensive, transgenerational alterations in cytosine methylation and to a less extent also genetic variation in Tong211-LP and its immediate progenies. *mPing *mobility was found to correlate with cytosine methylation alteration detected by MSAP but not with genetic variation detected by AFLP. Assay by q-RT-PCR of the steady-state transcript abundance of a set of genes encoding for the various putative DNA methyltransferases, 5-methylcytosine DNA glycosylases, and small interference RNA (siRNA) pathway-related proteins showed that, relative to the rice parental line, heritable perturbation in expression of 12 out of the 13 genes occurred in the mutator-phenotype and its sefled progenies.

**Conclusion:**

Transgenerational epigenetic instability in the form of altered cytosine methylation and its associated TE activity occurred in a rice mutator-phenotype produced by pollinating the rice stigma with pollens of *O. biennis*. Heritably perturbed homeostatic expression-state of genes involved in maintenance of chromatin structure is likely an underlying cause for the alien pollination-induced transgenerational epigenetic/genetic instability, and which occurred apparently without entailing genome merger or genetic introgression.

## Background

It is widely accepted that hybridization between genetically differentiated natural plant populations is a frequent phenomenon, which contributes to genome evolution, and can lead to speciation via allopolyploidy or at the homoploid level [1-6]. Apart from the properties of hybridization that can be explained by classical genetic mechanisms such as direct transfer and/or recombinatory generation of beneficial alleles, recent studies in both plant and animals have revealed that wide hybridization may generate variations by novel means such as rapid structural genomic changes, novel gene expression trajectories and epigenetic alterations, which apparently transgress Mendelian principles [1,7-17] One possible mechanism for the occurrence of non-Mendelian genomic and transcriptomic changes as a result of hybridization is *lato sensu *the "genomic shock" hypothesis proposed by McClintock [18].

Several lines of circumstantial evidence have suggested that hybridization-associated genetic and epigenetic instabilities may also be provoked in unsuccessful or "abortive" hybridizations between distant and sexually incompatible species. For example, it was found that random integration of uncharacterized DNA segments from unrelated sources into cultured animal cells, and introgression of multiple, tiny chromatin segments from a distantly related donor species into a recipient plant species may be mutagenic and induce genetic and epigenetic variations [19-23]. Although in these instances, the introgression of alien DNA or chromatin segments were automatically assumed as the causal factor for the induced instabilities, no direct link between the two events was ever established. In fact, a common observation emerged from these studies has indicated that the genomic loci underwent the changes are largely random both with regard to their chromosomal distribution and to nature of the changed sequences, thus argues against localized effects (e.g., insertional mutagenesis). Therefore, it remained a formal possibility that at least some of the detected non-Mendelian genetic and epigenetic mutations in these cases may not have been induced by the integration of DNA or chromatin segments *per se*; instead, they might have been the consequence of the process of genetic transfer (in animals) or alien pollination (in plants), which conceivably may constitute a kind of "biological stress" and elicit genetic and epigenetic instabilities, a scenario consistent with McClintock's "genomic shock" hypothesis [18].

In theory, it is possible that the process of pollination by pollens even from a remote and incompatible species may constitute a "biological stress" to the recipient parent in myriad ways. For example, metabolites including small signal molecules (e.g., nitric oxide and reactive oxygen species [24]) and various phytohormones of the alien pollens may enter stigma cells of the recipient species during their physical contacts; conceivably, this may induce physiological and biochemical mismatches of various kinds. Consequently, if the cellular machinery responsible for the constant fine-tuning of chromatin structure is compromised, then the occurrence of epigenetic and even genetic instability is almost inevitable. In this regard, the pollination by alien pollens from an incompatible species may bear mechanistic resemblance to pathogen attack wherein the pathogen's DNA or RNA usually does not integrate into the host genome, yet its interaction with the host may cause genetic and epigenetic instabilities in the latter. Indeed, it was documented recently in tobacco that pathogen infestation caused both general genetic instability (due to increased somatic recombination) and alteration in cytosine methylation at specific loci in the infected plants, and both of which are heritable to successive biological generations [25,26].

The aim of this study was to explore if pollination by alien pollens from an extremely remote and apparently incompatible plant species, which obviously would not generate genome merger or genetic introgressions, may still impose a "biological stress" to the recipient maternal genome, and induce heritable genetic and epigenetic instabilities.

## Results and discussion

### Identification of a mutator-phenotype

**Figure 1:**
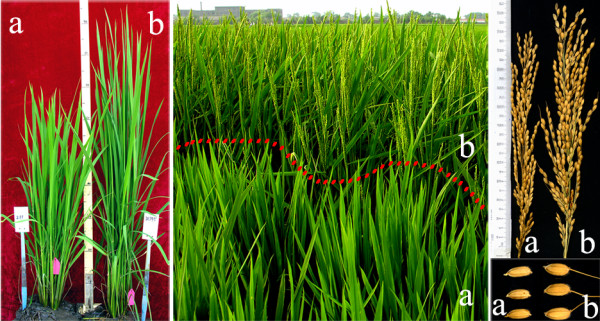
**Conspicuous phenotypic variation in multiple traits in selfed progenies (S1) of the single rice mutator-phenotype Tong211-LP after its rice parental line cv**. Tong211 was artificially pollinated by pollens of *Oenothera biennis *L. Leftmost and middle panels are overall plant statue of the rice parental line Tong211 (a) and S1 progenies of the mutator-phenotype Tong211-LP (b) at the vegetative and flowering stages, respectively. The rightmost panel exemplifies the panicle- and seed-sizes of the rice parental line Tong211 (a) and one individual of the S1 progeny of Tong211-LP (b).

We harvested more than 300 seeds from *ca*. 50 rice (*Oryza sativa *L. ssp. *japonica*, cv. Tong211) panicles that were artificially pollinated by fresh pollens taken from a single accession of a dicot plant, evening primrose (*Oenothera biennis *L.), followed by a second round pollination 48 hrs later with their own pollens collected from other individuals of Tong211 – a pollination method we termed "repeated pollination" [27]. We found that a substantial portion of the seeds showed abnormal development as a result of the manipulation, and which either did not germinate or died at early seedling stages. Among 84 rice plants (designated as S0 generation) growing to maturity, we identified a single individual plant (hereforth named Tong211-LP) that showed conspicuous phenotypic variation in multiple traits, including flowering time, seed-size, and particularly panicle-size (larger panicle, LP), relative to its rice parental cultivar. We then self-pollinated several panicles of this plant to produce seeds (also markedly enlarged), and the S1 progeny plants. Tong211-LP was partially sterile and produced a much smaller quantity of seeds than expected from a normal rice individual. Nonetheless, among the progenies produced, phenotypic variations that were observed in the individual mutator-phenotype (S0) were inherited at high frequencies to the S1 progenies; in addition, some new phenotypic variations that were apparently individual-specific appeared *de novo *in some of the S1 progeny plants. Typical phenotypic variations in the S1 progeny plants of the mutator-phenotype were shown in Figure 1. The transgenerational continuous occurrence of multiple phenotypic variations, i.e., sustained phenotypic instability, from a single individual is characteristic of a "mutator-phenotype", as originally reported in *Drosophila *[28].

### Transpositional activation of multiple TEs in the mutator-phenotype and its selfed progenies

**Figure 2:**
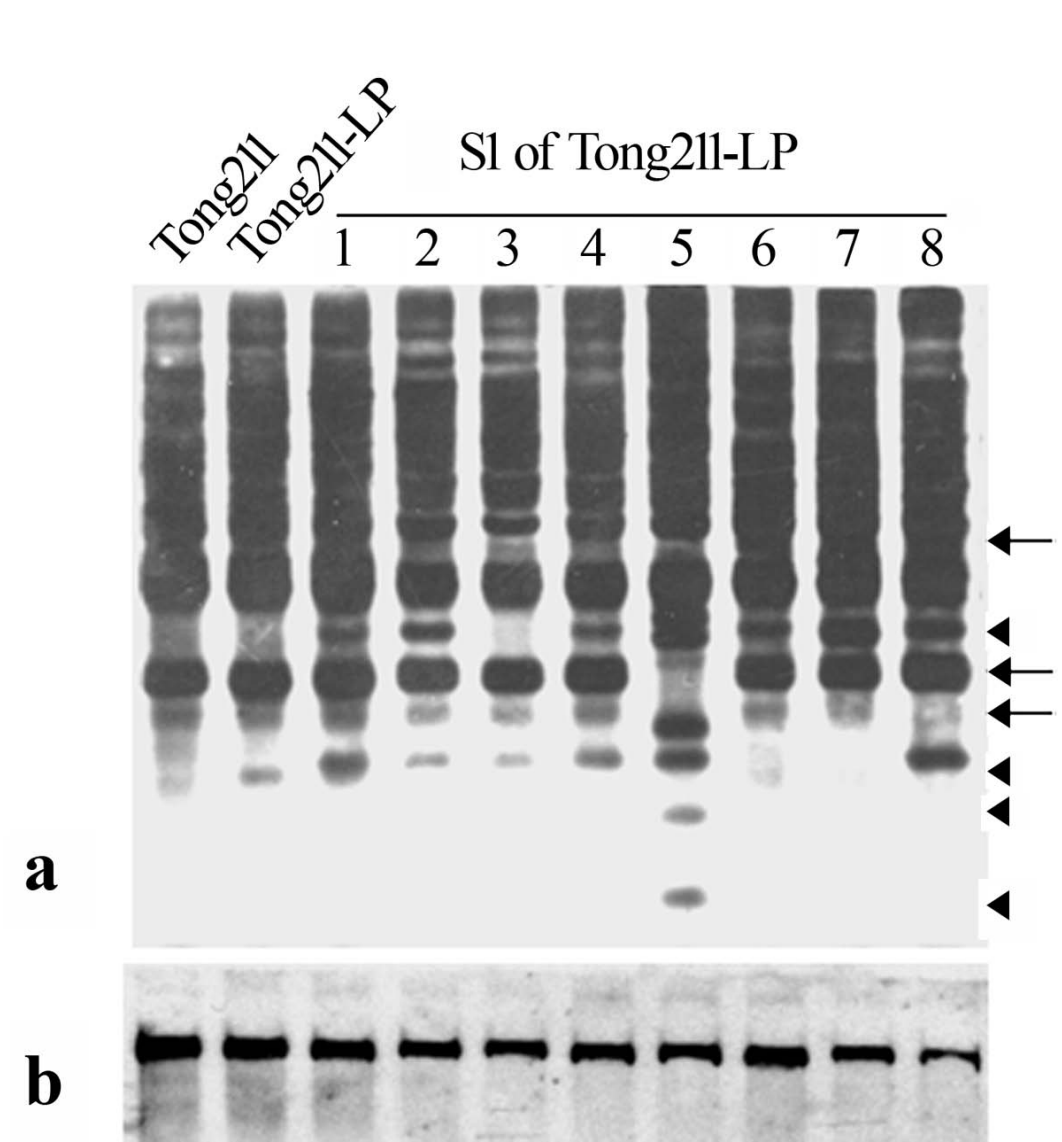
**Transpostional activation of *mPing *revealed by DNA-gel blotting**. (a) Hybridization of the full-length of *mPing *to a blot containing *Xba*I-digested genomic DNA of the various lines, including the rice parental line Tong211, the mutator-phenotype Tong211-LP and 8 individuals from its selfed progeny (S1). Arrows and arrowheads denote positions of loss and gain of bands in the S1 progenies of Tong211-LP, respectively. (b) Hybridization of a *Pong *fragment in the ORF2 region (*Pong*-ORF2) to the same blot as in (a). The monomorphic single band in all lanes indicates that these rice plants likely contain a single copy of *Pong*, and which was transpositionally static.

**Figure 3:**
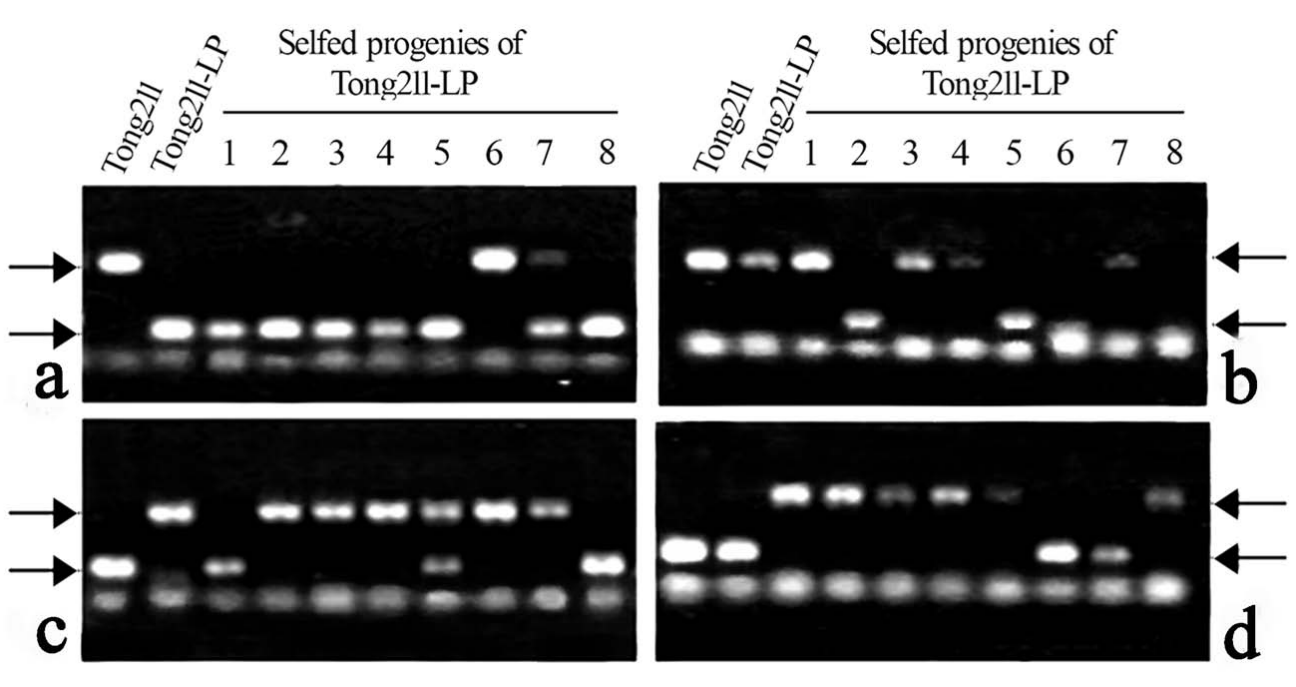
**Examples of validation of *mPing *excision and insertion events by locus-specific PCR amplifications**. PCR amplification products by using *mPing*-bracketing locus-specific primers on template DNA of the rice parent (Tong211), the mutant (Tong211-LP) and its 8 S1 progeny individual. (a) and (b) are excisions of *mPing *from the mutant Tong211-LP and/or some of its S1 progenies, while (c) and (d) are *de novo *insertions in the mutant Tong211-LP and/or some of its S1 progenies. Arrows refer to positions of the upper larger-sized bands and lower smaller-sized bands, the size difference between which is exactly 430 bp (the full-length of *mPing*), based on sequencing.

**Figure 4:**
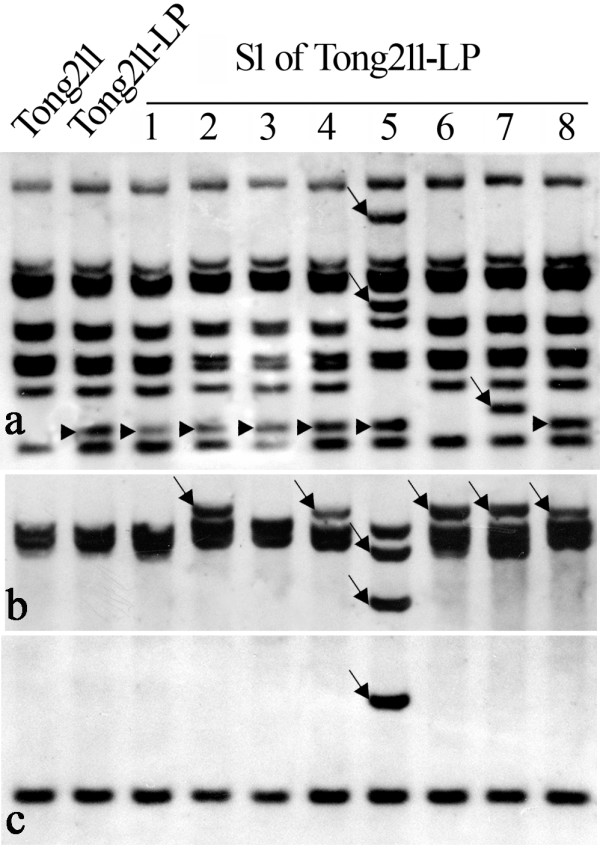
**Transpositional mobilization of three LTR-retrotransposons revealed by DNA-gel blotting**. (a), (b) and (c) are hybridization patterns for *Osr7*, *Tos17 *and *Osr23*, respectively. For each element, a portion of the reverse transcriptase (RT) region was amplified by specific primers (listed in Additional file 4) and used as a probe against the same blot as used in Figure 2. Arrows indicate novel bands appeared in the mutant Tong211-LP or its S1 progenies relative to their rice parent Tong211, which are suggestive of *de novo *retrotranspositional events. Note that progeny #5 showed the most rampant retrotranspositions, as is also the case for *mPing *(Figure 2); in addition, loss of bands was evident for two retrotransposons (*Osr7 *and *Tos17*) in this individual, suggesting the occurrence of genomic rearrangements within or adjacent to each of the element copies.

Genomic DNA was isolated from expanded young leaves of Tong211-LP (vegetatively propagated from root-stock cuttings), its eight selected S1 progeny individuals that exhibited the most extreme phenotypic variations, the parental rice line (cv. Tong211) and the pollen-donor, *O. biennis*. A series of DNA gel blot analysis was performed. First, to look for possible genetic introgression from the pollen donor *O. bienni *into rice, we did a genomic DNA probing assay [29], i.e., using labeled genomic DNA of *O. biennis *as a probe and autoclaved genomic DNA (150× excess in quantity) of rice as a blocker. We detected no evidence for genomic introgression (data not shown) based on this assay, as expected given the extreme phylogenetic remoteness of the two species. It should be pointed out however that this genomic blotting assay, though efficient [29], is by no means exclusive as only introgression of *O. biennis *species-specific genomic sequences would have been detected, and therefore cryptic introgression incidents may go undetected. Next, given the transgenerational mutability of multiple phenotypic traits, we asked the question if some normally quiescent transposable elements (TEs) might have been activated in the mutator-phenotype, as in the case of *Drosophila *[30,31], and in *bona fide *wide hybrids of plants [32]. We thus checked stability of several previously characterized low-copy TEs endogenous to the rice genome, which were either documented to be active under certain stress conditions [33-36], or were suspected so based on bioinformatic predictions [37]. The studied TEs included a 430 bp MITE, *mPing*[34], its two transposase donors, *Ping *and *Pong *[34,38], nine low-copy LTR retrotransposons, *Osr2, Osr3, Osr7, Osr23, Osr35, Osr36, Osr42*, *Tos19 *and *Tos17 *[33,37]. We found that four (*mPing*, *Osr7, Tos17 *and *Osr23*) of the 12 studied TEs showed apparent transpositional activation in Tong211-LP, or more often, in its S1 progenies (Figures 2, 3 and 4). A notable feature of the gel blotting patterns of these four TEs was that some of the S1 progenies exhibited individual-specific patterns, i.e., the pattern of a given individual was not shared by other sib-individuals, thus suggesting stochastic, transgenerational inheritance of the destabilized state in these elements, which mirrors the observation of phenotypic novelty in these plants Among the eight studied S1 individuals, #5 showed the most transpositions for all four elements (Figures 2, 3 and 4). It remained to be determined if this "transpositional active state" would be continually inherited to future generations, or it would be converted to a stabilized, repressive state after a few generations.

Two (*mPing *and *Tos17*) of these four elements were shown previously as active under various stressful conditions, including tissue culture [34,35] and irradiation [36], but the other two (*Osr7 *and *Osr23*) were only implicated as potentially active based on bioinformatic predictions but not empirically documented [37]. Thus, this is the first demonstration of transpositional activity of these two LTR retrotransposons in rice under this specific condition (Figures 4a and 4c).

Next, we studied the transposition of *mPing *in Tong211-LP and its S1 progenies in more detail, as this element is most active as well as amenable to characterization. First, the transpositional activity of *mPing *in these plants was further verified by transposon-display or TD [39], whereby > 60 putative *mPing *excision and *de novo *insertion events were isolated and sequenced. The sequence data indicated that at least 52 of the isolated events represent *bona fide mPing *activities (i.e., excisions or *de novo *insertions; see Additional files 1 and 2), as they each contained at their 5' terminus the expected portion of the *mPing *sequence encompassing the typical 15 bp terminal inverted repeats (TIRs) and the 3-bp target site duplication (TSD) of TAA or TTA, which is characteristic of the transpositional behavior of *mPing *[34-36]. Based on the sequence information, together with the complete genomic sequence of the standard laboratory rice genotype Nipponbare, locus-specific primers were designed for a set of 30 loci (see Additional files 1 and 2), which showed identical or high degree of sequence conservation between the studied rice cultivar (Tong211) and the genome-sequenced *japonica *rice cultivar Nipponbare. All these 30 loci were successfully amplified, cloned, sequenced and characterized (see Additional files 1 and 2). Sixteen of the 30 loci represent excisions in the S1 progenies of Tong211-LP, that is, compared with the parent Tong211 and the mutator-phenotype Tong211-LP (S0), they were excised in one or more of the S1 progenies (see Additional file 1). The predominant occurrence of *mPing *excisions in the S1 rather than the S0 generation (also evident in the gel blotting pattern, Figure 2) suggests that the timing of the excision events should be during late vegetative development and/or early gametogenesis in the Tong211-LP (S0) plant, and being manifested in the next generation via germline inheritance. Pairwise sequence comparison showed that none of 16 excisions had left behind any excision footprint (see Additional file 1), which is in agreement with some [38,40] but not all (e.g., [26,27]) previous reports on the excision properties of *mPing*. A BlastN analysis of these 16 excised *mPing *loci against the whole genome sequence of Nipponbare revealed an unexpected result in that eight of 16 loci were mapped to chromosome 3 and the rest to chromosomes 1, 2, 4, 11 and 12 (see Additional file 1), suggesting differential activity of the *mPing *copies with regard to their chromosomal locations.

Fourteen of the TD-identified loci were *mPing de novo *insertions in the S1 progenies of Tong211-LP, i.e., compared with the parent Tong211 and Tong211-LP (S0) they became larger-sized due to insertion of intact *mPing *copies in some of the S1 progenies (see Additional file 2). A BlastN analysis of these insertion-targeted sequences against the whole genome sequence of Nipponbare showed that all insertions mapped to unique- or low-copy regions (see Additional file 2), consistent with insertional propensity of *mPing *[41]. In contrast to the situation of excisions, these 14 *mPing *insertions did not show an obvious bias towards a particular chromosome (see Additional file 2).

### Genome-wide genetic and epigenetic instability in the mutator-phenotype

**Figure 5:**
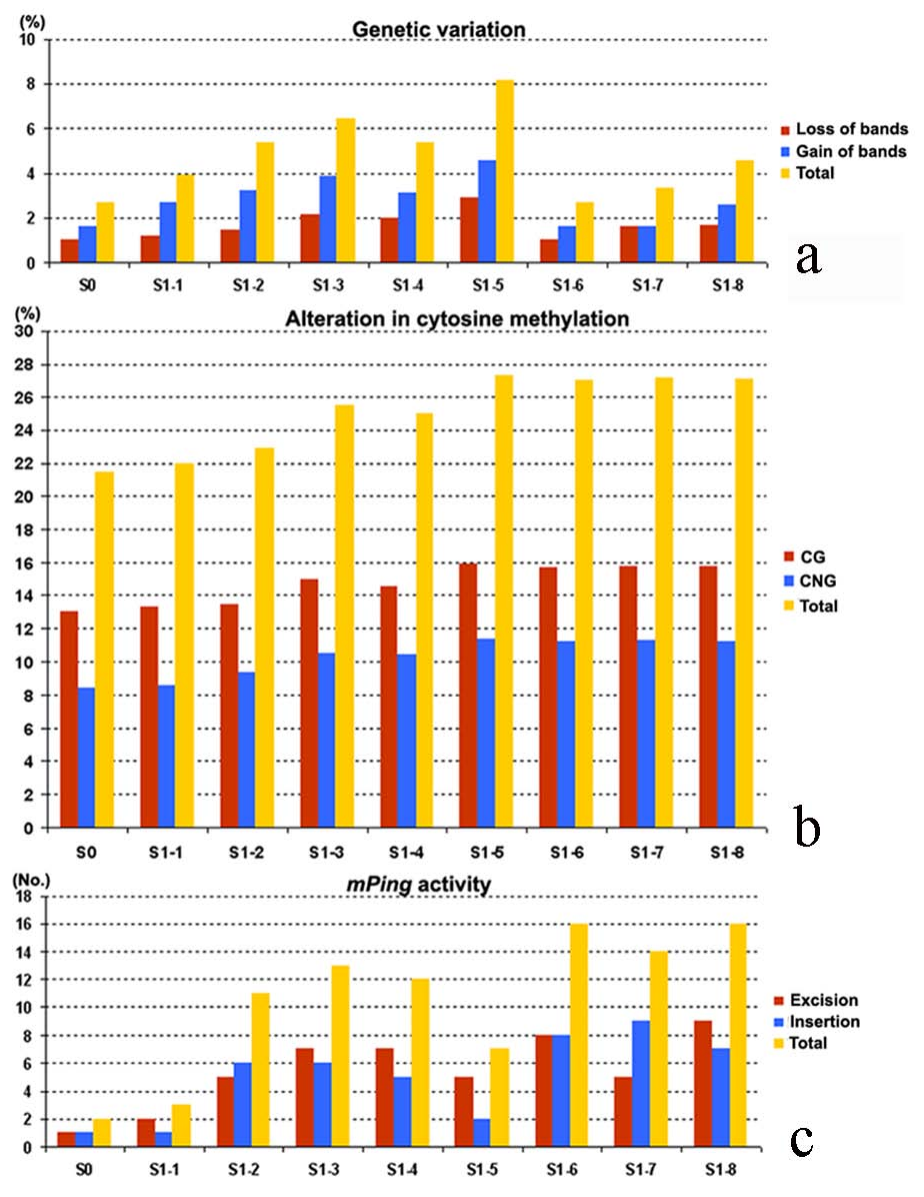
**Summary of the frequency or number of genetic changes detected by AFLP (a), alterations in cytosine methylation (at the CCGG sites) detected by MSAP (b) and *mPing *transpostional activity revealed by transposon-display (TD) (c), in the mutator-phenotype Tong211 (S0) and its 8 S1 progeny individuals**.

To test whether the genetic variations in Tong211-LP and its progenies were confined to the activation of a few specific TEs, or a more general genomic instabilities have been elicited, we performed genome-wide analysis on the same set of plants used in the gel-blotting by using AFLP and MSAP markers, and assessed > 1,000 loci for each marker. In the AFLP analysis, both loss of the rice parental bands and gain of novel bands were detected in Tong211-LP and its S1 progenies relative to their rice parent Tong211, with variation frequencies of both types of genomic changes together ranged from 2.66% to 8.16% (Figure 5). In MSAP analysis, methylation alteration of both CG and CNG at the CCGG sites (a prominent site for methylation modification in eukaryotes) were also detected in Tong211-LP and its S1 progenies relative to their rice parent Tong211, with frequencies of both types of DNA methylation alterations together ranged from 21.44% to 27.30% (Figure 5), which are higher by more than three times than those of genetic changes. These data indicated that, apart from transpositional activation of a subset of TEs (Figures 2, 3 and 4), pollination by pollens of *O. biennis *had particularly induced genome-wide epigenetic instabilities in the form of altered cytosine methylation patterns in the mutator-phenotype and its S1 progenies, though lower frequencies of genetic changes also occurred. It is notable that, as in the case for the transposition of the four TEs, described above, many loss or gain of bands in the AFLP or MSAP profiles were singletons (a specific change occurred in only one individual), thus probably could not be attributed to genetic segregation from existing variations in the S0 generation alone. Instead, stochastic transgenerational inheritance of the induced metastable epigenetic chromatin state might be a major underlying cause for the new variations in the S1 progeny, which is apparently consistent with the *de novo *appearance of phenotypic variations in the S1 plants (e.g., Figure 1).

To gain some insights into the chromosomal distribution and possible functional relevance of the genomic loci that showed genetic and epigenetic instabilities in Tong211-LP and its progenies, a set of variable AFLP and MSAP bands were isolated, cloned and sequenced (see Additional file 3). It was found that all variable bands were chromosomal DNA sequences of the rice genome (thus again pointing to the lack of genetic introgression from the pollen donor *O. biennis*), and they mapped to all 12 rice chromosomes, with the numbers ranged from two to six for each chromosome. A notable observation from the inferred possible functionalities of these variable bands is that the majority of them (23 out of 41) appeared to be genic sequences, followed by TEs (11), and only seven showed no homology (see Additional file 3). This may explain the dramatic phenotypic variations in the mutator-phenotype and its selfed S1 progenies (e.g., Figure 1).

### Correlations between two of the three kinds of instabilities, genetic variation, epigenetic variation, and TE activity

Given the concomitant occurrence of genetic variation, alteration in cytosine methylation and TE activity in the mutator-phenotype and its selfed progenies, it would be interesting to know whether these events are intrinsically correlated with, or independent of, each other. Thus, we calculated the Pearson's coefficients between each two of the three kinds of instabilities. We found no meaningful correlation between the genetic variation detected by AFLP and the alteration in cytosine methylation detected by MSAP (Table 1), suggesting the two kinds of variations were likely caused by independent mechanisms [42]. Likewisely, no correlation was seen between the genetic variation and *mPing *activity (including excision and insertion, detected by transposon-display) (Table 2), which is as expected given the known distinct cellular mechanisms for maintaining general genetic stability and control of TE activity. In contrast, statistically meaningful correlations were found between *mPing *activity and alteration in cytosine methylation (Table 2). Specifically, *mPing *excision is positively correlated with alteration in both CG and CNG methylation (P < 0.05 or 0.01), and *mPing *insertion is positively correlated with alteration in CNG methylation (P < 0.05) (Table 2). This is consistent with the genome-defense paradigm of cytosine methylation [43,44]. According to this paradigm, the primary role of cytosine methylation was evolved to serve as a genome defense system particularly to control mobility of endogenous TEs, and therefore stress-induced alteration in scope and/or extent of this epigenetic marker may provoke activation of otherwise quiescent TEs [43].

**Table 1:** Correlation between the genetic variations detected by AFLP and alteration in cytosine methylation detected by MSAP based on Pearson's coefficients

Genetic variation detected by AFLP	Alteration in cytosine methylation detected by MSAP	
	
	CG	CNG
Loss	0.488 (P_0.05 _= 0.183)	0.515 (P_0.05 _= 0.156)
Gain	0.123 (P_0.05 _= 0.753)	0.163 (P_0.05 _= 0.673)

**Table 2:** Correlations of *mPing *activity respectively with the genetic variations detected by AFLP and alteration in cytosine methylation detected by MSAP, based on Pearson's coefficients

*mPing *activity detected by TD	Genetic variation detected by AFLP		Alteration in cytosine methylation detected by MSAP	
	
	loss	gain	CG	CNG
excision	0.286 (P_0.05 _= 0.455)	0.166 (P_0.05 _= 0.670)	0.732* (P_0.05 _= 0.025)	0.800** (P_0.01 _= 0.010)
insertion	-0.075 (P_0.05 _= 0.848)	-0.286 (P_0.05 _= 0.456)	0.609 (P_0.05 _= 0.081)	0.675* (P_0.05 _= 0.046)

* and ** respectively indicate statistical significance at the 0.05 and 0.01 levels.

### Heritable alteration in expression state of genes encoding for putative DNA methyltransferase, 5-methylcytosine DNA glycosylase and siRNA pathway-related protein in the mutator-phenotype and its progenies

**Figure 6:**
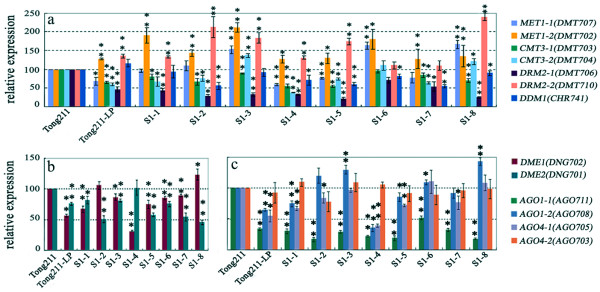
**Alteration in transcript abundance of a set (13) of genes encoding for putative DNA methyltransferases (a), 5-methylcytosine DNA glycosylases (b), and siRNA pathway-related proteins (c) in the mutator-phenotype (Tong211-LP) and 8 selected S1 individuals, relative to their parental rice line Tong211**. Real-time RT-PCR analysis on transcript quantity of the 13 genes in expanded young leaf tissue was performed on three batches of independent RNA-derived cDNAs with gene – specific primers (see Additional file 5). The relative abundance of transcripts (means ± SD) for each of the studied genes was calculated upon normalization against a rice β-actin gene (Genbank accession X79378). The gene names are labeled. * and ** denote statistical significance at the 0.05 and 0.01 levels, respectively.

Given that two known mechanisms, cytosine methylation and small interfering (si) RNA, often play critical roles in repressive control of TE activity [43-47], and the above documented correlation between alteration in cytosine methylation and TE (*mPing*) activity in the rice mutator-phenotype, it is reasonable to assume that the TE activity and epigenetic instability are probably related to perturbation of the homeostatic expression state of genes encoding for the enzymatic machinery responsible for maintaining the cytosine methylation states and/or other aspects of the chromatin epigenetic structure. We thus measured the mRNA steady-state abundance for a set of genes encoding for putative DNA methyltransferases, 5-methylcytosine DNA glycosylases, and the siRNA pathway-related proteins by real-time reverse transcriptase- PCR (q-RT-PCR) analysis with gene-specific primers. We found that of the 13 selected genes analyzed, 11 (the two exceptions are *DDM1 *and *AGO4-2*) showed significantly perturbed expressions in the mutator-phenotype (Tong211-LP) relative to its rice parental line (Tong211) (Figure 6). Furthermore, the perturbed expression states of these genes were transgenerationally heritable in the sense that none of the genes had reverted to the original expression states of their parental rice line (wild type) in all or most of the eight S1 plants analyzed (Figure 6). In fact, the extent of perturbation in the expression states of most of the genes was further augmented in some of the S1 progenies, and in one extreme case (i.e., the *DDM1 *gene), whose expression did not exhibit significant deviation from the parental line in Tong211-LP, nonetheless showed significant difference in six (except for S1-1 and -3) of the eight S1 individuals (Figure 6). This result of transgenerational perturbation in the expression states of the chromatin structure-maintenance genes is consistent with the heritable epigenetic instability in these plants, described above.

Recent studies have established that the intrinsic DNA methylation patterns in both plants and animals are faithfully maintained and perpetuated by coordinated function of at least two classes of DNA methyltransferases (maintenance and *de novo*), together with active demethylases, i.e., the 5-methylcytosine DNA glycosylases [48,49]. On the other hand, small interference RNA (siRNA) was documented as playing pivotal roles in repressing activity of TEs in diverse organisms by specific targeting [44-47]. Furthermore, at least in plants *de novo *methylation is often related to the activity of certain species of siRNAs by a mechanism known as RNA-directed DNA methylation or RdDM [50]. It is therefore conceivable that the coordinated expression of these genes represent a default requirement for stable maintenance and perpetuation of intrinsic DNA methylation patterns and silent TE states. Thus, the transgenerational perturbation of these genes in the mutator-phenotype and its progenies (Figure 6) may conceivably disrupt the homeostatic expression state of these genes as a network in the rice cells. It is likely that at least one facet of the possible effect of alien pollination as a "biological stress" may lie in its perturbation of coordinated expression of these chromatin state maintenance and regulation genes in the maternal recipient somatic and germinal cells, and hence, result in transgenerational epigenetic instability and TE activation.

### The mutator-phenotype was not caused by parental heterozygosity or contamination, but resultant from the alien pollination-imposed stress

There are three alternative possible causes that may be responsible for or contribute to the generation of the mutator-phenotype: one is segregation of pre-existing parental heterozygosity, the second is seed or pollen contamination from other rice cultivar(s), and the third is mutagenic effect from some unknown source. That we consider pollination by *O. biennis *as the only major underlying cause for the genetic and epigenetic instabilities in the mutator-phenotype (Tong211-LP) and its S1 progenies, are based on the following lines of evidence: First, the rice parental cultivar cv. Tong211 is a genetically pure line, as rice is a predominantly self-pollinating plant, and furthermore, the specific strain used for the present work had been maintained by strict selfing for > 10 successive generations in our hands, thus its inbred nature was ensured. In fact, the inbred nature of Tong211 has also been validated in this study by a parallel analysis on 30 random individuals, as in no case a variable pattern suggestive of heterozygosity was observed in either the gel-blotting patterns of the four active TEs or in PCR-based locus-specific *mPing *amplifications of all 30 loci (see Additional files 1 and 2; data not shown). Therefore, parental heterozygosity can be confidently ruled out as a causal factor for the markedly changing patterns of either the studied TEs, and by extension, the variable MSAP/AFLP profiles. Second, based on the following lines of evidence, contamination by pollens or seeds of other rice cultivars was considered as extremely unlikely. (1) Strict precautions were taken both in the cross manipulations (emasculation and pollination) and in later propagations by timely bagging of all panicles to endure 100% selfing. (2) It is notable that whereas most genetic and epigenetic changes that occurred in the mutator-phenotype were largely inheritable to its S1 progenies, many individual-specific new patterns appeared *de novo *in the S1 individuals (e.g., Figures 2, 3 and 4). Therefore, if contamination were a cause for the observed variable patterns, then the S1 plants need to have derived from S0 seeds that were contaminated independently by pollens of different rice cultivars, which obviously is extremely unlikely. (3) In the course of identifying the *mPing*-containing loci in cultivar Tong211, we uncovered 21 additional loci each contains a *mPing *copy in the standard cultivar Nipponbare but devoid the element in Tong211 (see Methods). We then amplified these 21 *mPing*-empty loci from the mutator-phenotype and its eight S1 progeny individuals, and we found that in all cases, only smaller-sized PCR products consistent with lacking of *mPing *in these plants were amplified (data not shown). Given the extremely high degree of presence *vs*. absence polymorphism of *mPing *among *japonica *rice cultivars [[34,51]; our unpublished data], this result strongly suggests that the mutator-phenotype and its analyzed progenies were unequivocally originated from one cultivar (Tong211) only. Taken together, the possibility of pollen or seed contamination can be confidently ruled out. Finally, all plant lines used in this study were grown together under identical normal conditions, under which biotic (e.g., pathogen infestation) and abiotic stresses were not exerted. Therefore, it is also inconceivable that the mutator-phenotype and its progenies had been differentially stressed from their parental line by an unknown stress that elicited the genomic instabilities.

By ruling out each of the three alternative possibilities, we are confident to conclude that pollination by *O. biennis *is the major, if not the only, conceivable cause for the genetic and epigenetic variations in the mutator-phenotype and its sefled progenies. Nonetheless, the basis for the occurrence of such extensive genetic and epigenetic instabilities as a result of "abortive" alien pollination remains mysterious. The single mutator-phenotype (Tong211-LP) was identified out of 84 "pollinated" plants, based on its striking phenotypic variations, thus giving a mutation frequency of 1.2%. Although it is likely that genomic variations may also have occurred in some other treated plants, they did not reach the extent to cause apparent phenotypic variations. Because all these 84 plants were sequentially pollinated first by *O. biennis *and then by pollens from the same rice line (Tong211), it appeared likely that stochasticity have also played an important part in the genesis of the mutator-phenotype individual.

The phenomenon we reported here is reminiscent of what McClintock envisioned two decades ago that wide hybridization in plants might activate quiescent TEs and cause genomic restructuring [18]. Indeed, several lines of empirical evidence in both plants and animals have lend support to this prediction [8,13,30-32,40,52-55]. Although all these previous works involved documented genome merger and/or introgression, it can be envisioned that even in the absence of introgression a "shock" at multiple levels may be incurred if the pollination by alien species *per se *represents a kind of "biological stress". In principle, even between incompatible crosses, certain metabolites, particularly those that require only trace quantity to produce dramatic effects like signal molecules, phytohormones and siRNA species etc., may be released from the donor pollens and enter the recipient stigma cells, thus may conceivably produce various mismatches and elicit a stress response.

It can be imagined that the cellular machinery responsible for safeguarding the genetic and epigenetic stabilities is likely sensitive as well as responsive to perturbations by stress, and fine-tuning on a balance between genetic/epigenetic fidelity and instability is required for the sake of survival and adaptation. Thus, in this study the significantly perturbed expression of nearly all of the 13 studied genes involved in the cellular machinery responsible for maintenance of chromatin epigenetic state subsequent to alien pollination might represent a sensory and adaptive response by the plant genome. From this perspective, the findings of this study may have bearing to genome evolution, as similar incidents of alien pollination may occur frequently under natural conditions, and hence, implicate a novel role of hybridization in evolution. Thus, we propose the possibility that "accidental cross-pollination" by a certain unrelated species may be actually mutagenic and elicited dramatic genetic/epigenetic instabilities, which may be perceived by selection. Further judiciously designed experiments involving an array of cross manipulations between different plant species are needed to investigate generality of this phenomenon and its underlying mechanism.

## Conclusion

To test the possibility that pollination by an unrelated and incompatible species may constitutes a "biological stress" whereby the genetic and epigenetic stability of the maternal parent genome might be jeopardized, we performed a crossing experiment between rice (served as the maternal partner) and *Oenothera biennis *L. (served as the pollen donor). A single rice mutator-phenotype individual (Tong211-LP) with conspicuous variation in multiple phenotypic traits was identified from the crossing experiment. Tong211-LP and its sefled progenies exhibited transgenerational epigenetic instability in the form of altered cytosine methylation and transpositional activation of several otherwisely quiescent transposable elements (TEs) endogenous to the rice genome. Heritably perturbed homeostatic expression-state of a set of genes involved in maintenance of chromatin structure is likely an underlying cause for the alien pollination-induced transgenerational epigenetic/genetic instability, and which occurred apparently without entailing genome merger or genetic introgression. Our results suggest that accidental pollination by unrelated alien pollens in plants might impose a stress condition and induce genetic and epigenetic instabilities in the maternal genome.

## Methods

### Plant materials

Plants used in this study included a single rice individual named "Tong211-LP" that was identified from a set of plants derived from seeds of a "alien pollination experiment" between rice (*Oryza sativa *L.), ssp. *japonica*, cv. Tong211 and a dicot plant, evening primrose (*Oenothera biennis *L.), and followed by self-pollination with pollens of different individuals of the same rice cultivar, namely, using a procedure we termed "repeated pollination" [27,40]. The choice for the particular crossing partners was based on two major considerations: (1) the two species (*O. sativa *L. and *O. biennis *L.) are hardly related, and hence, served well for the purpose of this study; (2) *O. biennis *L. produces a large amount of pollens that are viable for relatively long period after collection (our unpublished observation), and hence, convenient for the crossing manipulations. The identified individual plant (Tong211-LP) exhibited conspicuous phenotypic variation in multiple traits particularly enlarged panicles and seed-size, compared with its maternal parental cultivar Tong211. Seeds of individual panicles collected from Tong211-LP were selfed to produce the S1 progenies. In all cases, mutant plants were grown together with the parental line under identical, normal conditions, and strict bagging was practiced.

### DNA gel blot analysis

Genomic DNA was isolated from expanded young leaves of individual plants by a modified CTAB method [56] and purified by phenol extractions. Genomic DNA (~3 μg per lane) was digested by *Xba*I (New England Biolabs Inc.), and run through 1% agarose gels. The choice of *Xba*I is because the studied TEs either do not have a restriction site or the site(s) being on one side of the probe region, such that copy number of the TEs can be estimated based on the blotting patterns. Fractionated DNA was transferred onto Hybond N+ nylon membranes (Amersham Pharmacia Biotech) by the alkaline transfer recommended by the supplier. For investigating stability of a set of 13 low-copy transposable elements (TEs), element-specific primers were designed (see Additional file 4), and the fragments were obtained by PCR amplifications by using genomic DNA of the parental line (Tong211) as the template. The fragments representing each of the TEs were then gel-purified, identities confirmed by sequencing, and labeled with fluorescein-11-dUTP by the Gene Images random prime-labeling module (Amersham Pharmacia Biotech). Hybridization signal was detected by the Gene Images CDP-Star detection module (Amersham Pharmacia Biotech) after washing at a stringency of 0.2 × SSC, 0.1% SDS for 2 × 50 min. The filters were exposed to X-ray film for 1–3 hrs depending on signal intensity.

### Transposon-display (TD) and PCR-based locus assay on *mPing *excision and insertion

The transposon-display (TD) technique [39], using nested *mPing*-specific primers together with a primer designed according to the restriction enzyme *Mse*I-adapter sequence was as described [40]. To further verify *mPing *excisions and insertions, a subset of identified TD loci were sequenced, and by taking advantage of the complete genome sequence of the standard laboratory *japonica *rice cultivar Nipponbare , a set of locus-specific primers (see Additional file 1) each bracketing an intact *mPing *in the parental rice cultivar Tong211 (for detecting excision) or in the mutant Tong211-LP and its S1 progeny individual(s) (for detecting insertions), was designed by the Primer 3 software . Likewisely, a set of 21 loci each of which does not encompass a mPing copy in the parental cultivar Tong-211 was also identified in the course of TD analysis. Primers specific to this set of loci were also designed, and used to validate single genotypic origin of the mutant (Tong-211-LP) and its progenies. PCR amplification with these primer pairs were then conducted on the corresponding plant materials. The amplicons were visualized by ethidium bromide staining after electrophoresis through 2% agarose gels. All identified sites for *mPing *excisions (along with the corresponding element-containing donor sites) and *de novo *insertions were isolated and sequenced, such that the excision prosperities (e.g., to leave footprint or not) and characteristics (e.g., chromosomal location and potential functionality) of insertion-targeted sequences could be determined or inferred.

### AFLP and MSAP analysis

The protocols suitable for amplified fragment length polymorphism (AFLP) and methylation-sensitive amplified fragment (MSAP) in rice were exactly as reported [22,57]. For each marker, > 1,000 loci were scored. Typical bands representing genetic changes (AFLP) and cytosine methylation alterations (MSAP) in the mutant or its S1 progenies, as compared with their parental cultivar Tong211, were isolated, cloned and sequenced. Homology analysis was performed by BlastX at the NCBI website .

### Real-time Reverse transcriptase (RT)-PCR analysis

Isolation of total RNA and cDNA synthesis was essentially as reported [24]. Specifically, total RNA was isolated from expanded young leaves at the same developmental stage as that used for DNA isolation by the Trizol Reagent (Invitrogen), following the manufacturer's protocol. The RNA was treated with DNaseI (Invitrogen), reverse-transcribed by the SuperScriptTM RNase H-Reverse Transcriptase (Invitrogen), and subjected to q-RT-PCR analysis using gene-specific primers. The q-PCR experiments were performed using a Roche LightCycler480 apparatus (Roche Inc.) according to the manufacturer's instruction and SYBR Premix Ex Taq (Takara) as a DNA-specific fluorescent dye. The primers for all 13 studied genes encoding for putative DNA methyltransferases (seven), 5-methylcytosine DNA glycosylases (two) and siRNA-related proteins (four) were designed by the Primer 5 software (see Additional file 5). Expression of a rice β-actin gene (Genbank accession X79378) was used as internal control with the primer pairs 5'-ATGCCATTCTCCGTCTT-3' and 5'-GCTCCTGCTCGTAGTC-3'. Thermal cycling conditions consisted of an initial denaturation step at 95°C for 30 s, followed by 45 cycles of 15 s at 95°C and 1 min at 60°C. Three batches of independently isolated RNAs were used as technical replications. The melting curve analysis with the LightCycler480 together with 1.5% agarose gel electrophoresis of the products were used to ensure that right size product without significant background was amplified in the reaction. The relative amounts of the gene transcripts were determined using the Ct (threshold cycle) method, as described by the manufacturer's protocol. Data were analyzed by using the software provided by Roche Company and calculated by the 2-ΔΔCt method. Quantitative results were given as mean expression (means ± SD).

### Statistics

Statistical significance was determined using SPSS 11.5 for Windows  and analyzed by Independent-Samples student's t-Test. Specifically, correlations were tested for the data of variation frequencies calculated based on the three markers, Transposon (*mPing*)-display, MSAP and AFLP, by using the Pearson correlation analysis. The same program was used to test for the statistical significance of differences in the relative expression of the set of 13 genes in the mutator-phenotype (Tong211-LP) and its sefled progenies relative to that of the rice parental line (Tong211).

## Authors' contributions

HYW, YC, YYZ, YW, and FN carried out the laboratory experiments, analyzed the data and participated in drafting the manuscript. XCC and JHZ performed all pollination work and maintained plants. CMX and BL designed the study and finalized the manuscript. All authors read and approved the final manuscript.

## Supplementary Material

Additional file 1 **Characterization of *mPing *excisions**. A total of 16 loci that were excised from one or more of the 8 studied progeny individuals (from S_1_-1 to S_1 _– 8) of the mutator-phenotype Tong211-LP (S_0_) were identified by *mPing*-specific transposon-display (TD) and validated by cloning, sequencing, and locus-specific PCR amplification. Click here for file

Additional file 2 **Characterization of *mPing de novo *insertions**. A total of 14 *mPing de novo *insertion events which occurred in some of the selfed progeny individuals (from S_1_-1 to S_1 _– 8) of the mutator-phenotype Tong211-LP(S_0_) were identified by *mPing*-specific transposons-display (TD) and validated by cloning, sequencing and locus-specific PCR amplification. Click here for file

Additional file 3 **Characterization of variant AFLP and MSAP bands**. Chromosomal location and functional homology of isolated variant AFLP and MSAP fragments from the mutator phenotype Tong211-LP (S_0_) and/or its 8 selfed progeny individuals (from S_1_-1 to S_1 _– 8) were determined based on the reference genome sequence of cv. Nipponbare. Click here for file

Additional file 4 **Primers used to amplify the probe fragments of 12 low-copy and potentially active transposable elements (TEs) endogenous to the rice genome**. Authenticity of the amplicons were verified by sequencing. Click here for file

Additional file 5 **Real-time q-RT PCR primers used to measure the transcript quantity of a set of genes related to the chromatin epigenetic maintenance machinery**. Primers of a set of 13 genes encoding for the putative DNA methyltransferases, 5-methylcytosine DNA glycosylases and siRNA-related proteins were designed by the Primer 5 software, based on the sequence information deposited at the Chromatin database . Click here for file
