# Supplementary material for: Molecular characterization of a rice mutator-phenotype derived from an incompatible cross-pollination reveals transgenerational mobilization of multiple transposable elements and extensive epigenetic instability
Source: BMC Plant Biol. 2009 May 29;9:63. doi: 10.1186/1471-2229-9-63 (PMC2696445; doi:10.1186/1471-2229-9-63)
Supplement: Additional file 1 — Characterization of mPing excisions. A total of 16 loci that were excised from one or more of the 8 studied progeny individuals (from S1-1 to S1 – 8) of the mutator-phenotype Tong211-LP (S0) were identified by mPing-specific transposon-display (TD) and validated by cloning, sequencing, and locus-specific PCR amplification. [file 1471-2229-9-63-S1.doc]

**Additional file 1** Characterization of *mPing* excisions at 16 loci in some of the selfed progeny individuals (from S_1_-1 to S_1_--8) of the mutator-phenotype Tong211-LP (S_0_), which were identified by *mPing*-specific transposon display (TD) and validated by cloning, sequencing, and locus-specific PCR amplification

| Excision  Locus | Excision  Site | Locus-specific primers  (5’-3’) | Excised from | Excision Footprint* |
| --- | --- | --- | --- | --- |
| TDE-1 | Chr.3  [AP008207.1](http://www.ncbi.nlm.nih.gov/entrez/query.fcgi?cmd=Retrieve&db=Nucleotide&list_uids=58530787&dopt=GenBank&RID=Y3MDFHHY013&log$=nucltop&blast_rank=1) | gctcgtggctgaagacctta  tcgtctctcggtgacacagt | S_1_-5 | tttgcatgcaTTA<*mPing*(430bp)>TTAcatgaagcaggt  tttgcatgcaTTA---------------------- ---catgaagcaggt |
| TDE-2 | Chr.1  AP008207.1 | gaaactaacgcgtgcacaga  gcgattcagcataacaccaa | S_1_-2, -3, -4, -5, -6, -8 | caaccctgtaTAA<*mPing*(430bp)>TAAgcatctttgt  caaccctgtaTAA--------------------------gcatctttgt |
| TDE-3 | Chr.3  AP008209.1 | tcccattcaaagatgacgaa gaacacgaaacaacagaacc | S_1_-6, -7 | tgcactttgcTTA<*mPing*(430bp)>TTAggggtgatca  tgcactttgcTTA--------------------------ggggtgatca |
| TDE-4 | Chr.11  [AL732353.1](http://www.ncbi.nlm.nih.gov/entrez/query.fcgi?cmd=Retrieve&db=Nucleotide&list_uids=22091858&dopt=GenBank&RID=Y3MSYSNM011&log$=nucltop&blast_rank=1) | gggctccacgtcatcataa  tcagctccaccaaaactgg | S_1_ -6, -7 | taggagagagTTA<*mPing*(430bp)>TTAtagcgtgtgc  taggagagagTTA--------------------------tagcgtgtgc |
| TDE-5 | Chr.4  CR855115.1 | ctgcacgcctagcctcttta  agcgctcgactactccagat | S_1_-1, -2, -3, -4, -5, -8 | ttcacgctccaTTA<*mPing*(430bp)>TTAgagcgctccg  ttcacgctccaTTA--------------------------gagcgctccg |
| TDE-6 | Chr.1  AP008207.1 | cgaatgcatcgataccactta taatggcccaattcaatgct | S_1_-2, -5, -6 | gacttaaataTAA<*mPing*(430bp)>TAAggcatgcatg  gacttaaataTAA--------------------------ggcatgcatg |
| TDE-7 | Chr.3  AC133333.5 | catgtgcgtggaaaacagag ggtgcggaacatgtcatcta | S_1_-6, -7 | aaacgcacccTTA<*mPing*(430bp)>TTAggctgagttt  aaacgcacccTTA--------------------------ggctgagttt |
| TDE-8  -1 | Chr.11  AP008217.1 | gccgcgagctaatgatagtt  gtaaccctgccctgactcat | S_1_-6, -8 | ataacaatgatAAT<*mPing*(430bp)>AATagccgttaaca  ataacaatgatAAT--------------------------agccgttaaca |
| TDE-8  -2 | Chr.5  AP008211 | tttgcagctggcttatagca  tcctcggtttagtcctgacg | S_1_-3, -8 | aggaggatctgTTA<*mPing*(430bp)>TTAgactttgttca  aggaggatctgTTA--------------------------gactttgttca |
| TDE-15 | Chr.2  [AP008208.1](http://www.ncbi.nlm.nih.gov/entrez/query.fcgi?cmd=Retrieve&db=Nucleotide&list_uids=58530788&dopt=GenBank&RID=Y3MX8U34015&log$=nucltop&blast_rank=1) | ggggagttgcaagtgttgat  tggtagttgcatccgattctt | S_1_-8 | ttcttgtcttcTAA<*mPing*(430bp)>TAAagaattttcct  ttcttgtcttcTAA--------------------------agaattttcct |
| TDE-18 | Chr.12  BX000506 | ttgagcgagagaaattaatcc  gccagttctttccgattgac | S_1_-3, -4, -6, -7, -8 | gatccaacgccTAA<*mPing*(430bp)>TAAgacatcatcac  gatccaacgccTAA--------------------------gacatcatcac |
| TDE-24 | Chr.3  AP008209 | gtcacaatgggggtttcact  tgaacctattccactcgaagaa | S_1_-2 | tggccggtgaaTTA<*mPing*(430bp)>TTAgtaccaagtcg  tggccggtgaaTTA--------------------------gacatcatcac |
| TDE-26 | Chr.3  AC133333 | gtcacaatgggggtttcact  atgctgtggctttggattct | S_1_-3, -4 | aaaactcagccTAA<*mPing*(430bp)>TAAgggtgcgtttg  aaaactcagccTAA--------------------------gggtgcgtttg |
| TDE-32 | Chr.3  AP008209 | tgggcttaacctagacgtagaa  tgtaatagtttgggggtagttcg | S_1_-8 | gattcggttaaTTA<*mPing*(430bp)>TTAggacgttatcc gattcggttaaTTA--------------------------ggacgttatcc |
| TDE-33 | Chr.3  AC134887 | tggggatgagagagaaggaa  gatgaaagggcaccatttgt | S_1_-4 | cttgatcaccccTAA<*mPing*(430bp)>TAAgcaaagtgcac cttgatcaccccTAA--------------------------gcaaagtgcac |
| TDE-37 | Chr.3  AP008209 | gtcacaatgggggtttcact  tgaacctattccactcgaagaa | S_1_-3, -4, -8 | ttggccggtgaaTTA<*mping*(430bp)>TTAgtaccaagtcg ttggccggtgaaTTA--------------------------gtaccaagtcg |
