# Supplementary material for: Molecular characterization of a rice mutator-phenotype derived from an incompatible cross-pollination reveals transgenerational mobilization of multiple transposable elements and extensive epigenetic instability
Source: BMC Plant Biol. 2009 May 29;9:63. doi: 10.1186/1471-2229-9-63 (PMC2696445; doi:10.1186/1471-2229-9-63)
Supplement: Additional file 2 — Characterization of mPing de novo insertions. A total of 14 mPing de novo insertion events which occurred in some of the selfed progeny individuals (from S1-1 to S1 – 8) of the mutator-phenotype Tong211-LP(S0) were identified by mPing-specific transposons-display (TD) and validated by cloning, sequencing and locus-specific PCR amplification. [file 1471-2229-9-63-S2.doc]

**Additional file 2**  Characterization of *mPing* *de novo* insertions in some of the selfed progeny individuals (from S_1_-1 to S_1_--8) of the mutator-phenotype Tong211-LP(S_0_), which were identified by *mPing*-specific transposon display (TD) and validated by cloning, sequencing and locus-specific PCR amplification

| Insertion  Locus | Insertion  Site | Locus-specific primers (5’-3’) | Isolated  from | TIR | TSD |
| --- | --- | --- | --- | --- | --- |
| TDI-1 | Chr. 7  [AP008210.1](http://www.ncbi.nlm.nih.gov/entrez/query.fcgi?cmd=Retrieve&db=Nucleotide&list_uids=58530790&dopt=GenBank&RID=Y3N7EGJD011&log$=nucltop&blast_rank=1) | gcacaggctccaagacgta aaaaactgaccgttggatgg | S_1_-2, -3, -4, -6, -7 | ggccagtcacaatgg | TTA |
| TDI-3 | Chr. 4  [AP008207.1](http://www.ncbi.nlm.nih.gov/entrez/query.fcgi?cmd=Retrieve&db=Nucleotide&list_uids=58530787&dopt=GenBank&RID=Y3NAYKFV011&log$=nucltop&blast_rank=1) | ggcaatggtgattcgttga  tgcatgagagccaatactcc | S_1_-7 | ggccagtcacaatgg | TAA |
| TDI-7 | Chr. 3  AP008209 | aaaaatgaaccggggaaaac  ttgcatctgcctgtacttcg | S_1_ -8 | ggccagtcacaatgg | TTA |
| TDI-9 | Chr. 9  AP008215 | attaaatttgctctaatccggtca  ccagctgcagcacgaact | S_1_ -4, -8 | ggccagtcacaatgg | TAA |
| TDI-11 | Chr. 8  AP008214 | actctgctcgatcctcctca  gtgtttgatgaccgggagat | S_1_ -8 | ggccagtcacaatgg | TAA |
| TDI-13 | Chr. 2  AP008208 | gtgtttgatgaccgggagat  ccattgttggtagttgcatcc | S_1_-2, -3, -7 | ggccagtcacaatgg | TAA |
| TDI-20 | Chr. 1  [AP008207.1](http://www.ncbi.nlm.nih.gov/entrez/query.fcgi?cmd=Retrieve&db=Nucleotide&list_uids=58530787&dopt=GenBank&RID=Y3N2PJHE011&log$=nucltop&blast_rank=1) | atttaaaacacatcgttccaca  cgaatgcatcgataccactta | S_1_ -2 | ggccagtcacaatgg | TTA |
| TDI-21 | Chr. 2  AP008208 | gagaatggcttgggctaaca  gtgacgcgtctcccattatt | S_1_-3, - 4,  -6, -7, -8 | ggccagtcacaatgg | TAA |
| TDI-23 | Chr. 6  AP008212 | aaggggattgaggagattgg  ccaatgcactgagtagaaagga | S_1_-2 | ggccagtcacaatgg | TTA |
| TDI-25 | Chr. 2  AP008208 | tgcttatggtgcatccttttc  tgcttatggtgcatccttttc | S_1_-3, -4,  -6, -7, -8 | ggccagtcacaatgg | TAA |
| TDI-27 | Chr. 2  AP005012 | catgtcttgtttggggacaa  gtgccacccttcacaatgtc | S_1_-6 | ggccagtcacaatgg | TTA |
| TDI-29 | Chr. 3  AC084405 | aatcgaaccatcgctttacg  tggcccacttatcctttgac | S_1_-6, - 7 | ggccagtcacaatgg | TAA |
| TDI-38 | Chr. 8  AP008214 | tggcatatctgggcaaattc  ttatcctgatggcctcatcc | S_1_-5, -6, -7 | ggccagtcacaatgg | TTA |
| TDI-41 | Chr. 3  AP008209 | atttgctcgtggctgaagac  tccacctgttaagtttgtttcttg | S_1_-2, -3,  -6, -7, -8 | ggccagtcacaatgg | TTA |
