# Supplementary material for: Molecular characterization of a rice mutator-phenotype derived from an incompatible cross-pollination reveals transgenerational mobilization of multiple transposable elements and extensive epigenetic instability
Source: BMC Plant Biol. 2009 May 29;9:63. doi: 10.1186/1471-2229-9-63 (PMC2696445; doi:10.1186/1471-2229-9-63)
Supplement: Additional file 3 — Characterization of variant AFLP and MSAP bands. Chromosomal location and functional homology of isolated variant AFLP and MSAP fragments from the mutator phenotype Tong211-LP (S0) and/or its 8 selfed progeny individuals (from S1-1 to S1 – 8) were determined based on the reference genome sequence of cv. Nipponbare. [file 1471-2229-9-63-S3.doc]

**Additional file 3** Chromosomal location and homology of isolated variant AFLP and MSAP fragments from the mutator phenotype Tong211-LP (S_0_) and/or its eight selfed progeny individuals (from S_1_-1 to S_1_--8)

| Variable AFLP  or MSAP bands Sequenced | Length  (bp) | Plant(s) showing variation/Type of variation* | Chr.  location | Putative function |
| --- | --- | --- | --- | --- |
| AF-16 | 483 | S_1_-8/loss | 9 | ABA98926; retrotransposon protein, putative,  Ty3-gypsy subclass [Oryza sativa (japonica cultivar-group)]; 0 |
| AF-17 | 401 | S_1_-2, -3, -8/gain | 2 | Os01g29180;12001.t02608; unspliced- genomic expressed protein; e-160 |
| AF-18 | 396 | S_1_-5/gain | 1 | Os01g29180;12001.t02608; unspliced- genomic expressed protein; 0 |
| AF19 | 211 | S_1_-5/loss | 12 | Os11g43330;12011.t03863; unspliced-genomic conserved hypothetical protein; 1e-025 |
| AF-20 | 224 | S_1_-5, -6, -7/gain | 9 | Os09g25500;12009.t02229;unspliced-  genomic expressed protein; 3e-042 |
| AF-21 | 148 | S_1_-5/gain | 2 | Os02g09100;12002.t00757; unspliced-genomic isoleucine-tRNA ligase-like protein; 4e-037 |
| AF-22 | 128 | S_1_-5/loss | 8 | none |
| AF-23 | 267 | S_1_-1, -5/loss | 10 | Os10g35190;12010.t02814;unspliced-  genomic expressed protein; e-130 |
| AF-24 | 232 | S_0_, S_1_-1, -2, -3, -4, -5, -8/loss | 7 | Os01g33764;12001.t006885; unspliced-genomic expressed protein; 9e-018 |
| AF-25 | 288 | S_1_-5/gain | 10 | Os10g37899;12010.t03047; unspliced- genomic transposon protein, putative; e-122 |
| AF-27 | 345 | S_1_-5/loss | 3 | AAV31288; putative transposon polyprotein  [Oryza sativa (japonica cultivar-group)]; 9e-26 |
| AF-28 | 216 | S_1_-5, -6, -7/loss | 10 | Os10g37880;12010.t03046; unspliced-genomic  flavonol synthase/flavanone 3-hydroxylase,  putative, expressed; 7e-080 |
| AF-29 | 201 | S_0_, S_1_-1, -2, -3, -4, 8/loss | 9 | Os05g31290;12005.t02725; unspliced- genomic  acyl carrier protein, mitochondrial precursor,  putative, expressed; 2e-037 |
| AF-30 | 116 | S_1_-1, -3, -4, -5,-6, -7, 8/loss | 3 | none |
| AF-31 | 176 | S_1_-5/loss | 6 | none |
| AF-33 | 269 | S_1_-5/loss | 7 | Os02g35950;12002.t03233; unspliced-genomic expressed protein; 7e-019 |
| AF-34 | 193 | S_1_-5/loss | 4 | ABA96179; retrotransposon protein, putative, unclassified [Oryza sativa (japonica cultivar-group)]; 8e-21 |
| AF-35 | 240 | S_1_-2, -3, -4, -6, -7, 8/loss | 2 | EAZ22532; hypothetical protein OsJ_006015  [Oryza sativa (japonica cultivar-group)]; 3e-06 |
| AF-36 | 181 | S_1_-5/loss | 1 | AAS07056;retrotransposon protein, putative,  Ty3-gypsy sub-class [Oryza sativa (japonica cultivar-group)]; 6e-14 |
| AF-38 | 402 | S_1_-2, -3, -4/loss | 12 | none |
| AF-39 | 757 | S_0_, S_1_-2, - 3, -4, -5, 8/loss | 11 | AAX95354; NB-ARC domain, putative  [Oryza sativa (japonica cultivar-group)]; 1e-77 |
| AF-40 | 123 | S_0_, S_1_-2, -3, -4, -5, 8/loss | 2 | Os02g16300;12002.t01423; unspliced- genomic retrotransposon protein, putative, unclassified; e-153 |
| AF-42 | 115 | S_1_-6, -7/loss | 2 | none |
| AF-43 | 43 | S_1_-2, -3, -4/loss | 1 | EAZ10178; hypothetical protein OsJ_000003  [Oryza sativa (japonica cultivar-group)]; 4e-38 |
| AF-44 | 328 | S_1_-7/loss | 2 | EAY87710; hypothetical protein OsI_008943  [Oryza sativa (indica cultivar-group)]; 2e-55 |
| AF-45 | 154 | S_1_-7/loss | 12 | Os02g16300;12002.t01423;unspliced-genomic retrotransposon protein, putative, unclassified;  4e-056 |
| AF-46 | 196 | S_1_-1, -5/loss | 5 | CAE02781; OSJNBa0011L07.5 [Oryza sativa  (japonica cultivar-group)]; 4e-13 |
| MS-1 | 384 | S_1_-1/gain in both H & M | 4 | EAZ30603;hypothetical protein OsJ_014086  [Oryza sativa (japonica cultivar-group)]; 6e-13 |
| MS-2 | 346 | S_1_-1, -2, -3, -4, -5, 8/loss in M | 6 | BAD45607; putative UDP- glucuronyltransferase-l [Oryza sativa (japonica cultivar-group)];1e-29 |
| MS-3 | 275 | S_0_, S_1_-1, -2, -3, -4, -5, -8/ loss in both H & M | 9 | BAB33421;putative senescence-associated protein [Pisum sativum]; e-109 |
| MS-4 | 216 | S_0_, S_1_-1, -3, -4/loss in M | 12 | ABA94403; retrotransposon protein, putative, unclassified [Oryza sativa (japonica cultivar-  group)]; 4e-23 |
| MS-5 | 236 | S_0_, S_1_-1, -3/loss in both H & M | 12 | Os12g41570;12012.t03832; unspliced-genomic conserved hypothetical protein; 5e-013 |
| MS-6 | 191 | S_1_-5/loss in M | 11 | ABA92247; retrotransposon protein, putative,  Ty3-gypsy subclass [Oryza sativa (japonica cultivar-group)]; 5e-26 |
| MS-7 | 176 | S_1_-1, -5/loss in M | 11 | Os11g19700;12011.t01740; unspliced- genomic cycloeucalenol cycloisomerase, putative,  expressed; 3e-075 |
| MS-8 | 135 | S_0_, S_1_-2, -3, -4, -8/ loss in both H & M | 1 | Os01g57470;12001.t05151;unspliced-  genomic caltractin, putative, expressed; 2e-039 |
| MS-9 | 280 | S_1_-5/gain in M | 5 | ABA95227 retrotransposon protein, putative, unclassified [Oryza sativa (japonica cultivar-  group)]; 5e-42 |
| MS-11 | 116 | S_1_-5/gain in H | 8 | none |
| MS-12 | 572 | S_1_-6, -7/loss in M | 9 | Os06g25960;12006.t02403; unspliced- genomic hypothetical protein; e-172 |
| MS-13 | 186 | S_1_-5/gain in H;  S_0_, S_1_-1, -2, -3, -4, -5, -8/ loss in M | 8 | Os05g25410;12005.t02197;unspliced-  genomic retrotransposon protein, putative,  unclassified; 2e-006 |
| MS-14 | 181 | S_1_-6, -7/loss in both H & M | 6 | none |
| MS-15 | 125 | S_0_, S_1_-1, -2, -3, -4, -6, -7, -8/ gain in both H & M | 1 | Os01g04490;12001.t00337;unspliced-genomic  Ser/Thr protein kinase, putative, expressed; 1e-049 |

*Note: In AFLP: loss –- absence of rice parental band, gain—appearance of novel band, in Tong211-LPand/or its selfed progenies; in MSAP: loss and gain of band may occur in either or both of the *Hpa*II + *Eco*RI (H) digest and the *Msp*I + *Eco*RI (M).
