# Supplementary material for: Molecular characterization of a rice mutator-phenotype derived from an incompatible cross-pollination reveals transgenerational mobilization of multiple transposable elements and extensive epigenetic instability
Source: BMC Plant Biol. 2009 May 29;9:63. doi: 10.1186/1471-2229-9-63 (PMC2696445; doi:10.1186/1471-2229-9-63)
Supplement: Additional file 4 — Primers used to amplify the probe fragments of 12 low-copy and potentially active transposable elements (TEs) endogenous to the rice genome. Authenticity of the amplicons were verified by sequencing. [file 1471-2229-9-63-S4.doc]

**Additional file 4** Primers used to amplify the probe fragments of 12 low-copy and potentially active transposable elements (TEs) in rice

| Probe name | GenBank  Access. No. | Nucleiotide positions | Primer sequences |
| --- | --- | --- | --- |
| *mPing* | AB087615 | 6-430 | forward: 5’-GTCACAATGGGGGTTTCACT  reverse: 5’-GGCCAGTCACAATGGCTAGT |
| *Pong-ORF2* | BK000586 | 3199-4255 | forward: 5’- AACGAGGCTTCTGACCATCG  reverse: 5’- CAGGTTCCTGAACGGTTGAT |
| *Ping-specific* | AB087616 | 327-1513 | forward: 5’- CTACGGAGTACACCGCAACC  reverse: 5’- AATGGATTGCCTACTGCTGACT |
| *Osr2* | AL442110 | 2918-4417 | forward: 5’-CACACCAGCACCAAGTCCTA  reverse: 5’-TCGATCGCTTTAGGTTGCTT |
| *Osr3* | AF458765 | 3470-4963 | forward: 5’- ATCGACATACAGGGCCTTTG  reverse: 5’-TCAGCAACTTGTCCACCAGA |
| *Osr7* | AP002538 | 1628-2236 | forward: 5’- AGAGCCCGGTTAAGTTCGTT  reverse: 5’- AGCTTGTCCATGGTAAGGTCA |
| *Osr23* | AP002843 | 778-1973 | forward: 5’- GCCGGTCTTGATGATGAGTT  reverse: 5’-TTGAACAGACGCTCCACAAG |
| *Osr35* | AC068924 | 4232-5232 | forward: 5’- TGATGTGGTCCTTGAGTCCA  reverse: 5’- ATTCTCTTGGCTTGGCTGTG |
| *Osr36* | AP001551 | 3455-4655 | forward: 5’- CCCTGAATCCACCAAGAAAA  reverse: 5’- GGCAGTCTCGAGAAGGTGAC |
| *Osr42* | AF458768 | 4361-5240 | forward: 5’- CCACAGATCATCATTTCTGACC  reverse: 5’-CCCCTTGAAGACTGACTTGC |
| *Tos19* | T03721 | 563-1770 | forward: 5-TCTCAGCCTCCCACTTGTCT  reverse: 5’-TGTCCCATTGCCACATCTAA |
| *Tos17* | D85865 | 2817-3483 | forward: 5’-GCTACCCGTTCTTGGACTAT  reverse: 5’- CTGAAATCGGAGCACTGACA |
