# Supplementary material for: Molecular characterization of a rice mutator-phenotype derived from an incompatible cross-pollination reveals transgenerational mobilization of multiple transposable elements and extensive epigenetic instability
Source: BMC Plant Biol. 2009 May 29;9:63. doi: 10.1186/1471-2229-9-63 (PMC2696445; doi:10.1186/1471-2229-9-63)
Supplement: Additional file 5 — Real-time q-RT PCR primers used to measure the transcript quantity of a set of genes related to the chromatin epigenetic maintenance machinery. Primers of a set of 13 genes encoding for the putative DNA methyltransferases, 5-methylcytosine DNA glycosylases and siRNA-related proteins were designed by the Primer 5 software, based on the sequence information deposited at the Chromatin database . [file 1471-2229-9-63-S5.doc]

**Additional file 5** q-RT PCR primers used to amplify the 13 studied genes encoding for putative DNA methyltransferases , 5-methylcytosine DNA glycosylases and siRNA-related proteins

| Gene name | ChromDB* ID | Nucleiotide positions | Primer sequences |
| --- | --- | --- | --- |
| *MET1-1* | *DMT707* |  | forward: 5-CAGGTGGATTCAGATAAATGTA  reverse: 5’-AATCGCCCACTTTGTAAACG |
| *MET1-2* | *DMT702* |  | forward: 5-GAAGAAGATTAGAAGCGA  reverse: 5’-CTCCCAGTCCAACCTACC |
| *CMT3-1* | *DMT703* |  | forward: 5-GGTTTCGTGGAACAGTCAA  reverse: 5’-GGCGGCAGCAACATAAGG |
| *CMT3-2* | *DMT704* |  | forward: 5-GGAGCCTATTGAGAACCT  reverse: 5’-TGAACCGATTGAAACCAC |
| *DRM2-1* | *DMT706* |  | forward: 5-CCAAGGAGGCAGTTCAA  reverse: 5’-CATTCGTCCAAGACATAC |
| *DRM2-2* | *DMT710* |  | forward: 5-ACACCAGGCATCCGTAG  reverse: 5’-ATGTAACCCTCCTTTCG |
| *DDM1* | *CHR741* |  | forward: 5-GGGCTGGTGGGCTGGGTAT  reverse: 5’-GCATTAGGTTTGGCTCTGTCT |
| *DME1* | *DNG702* |  | forward: 5-ACTGGAAACGCTGTCG  reverse: 5’-CTGCTGCCAATGTCTT |
| *DME2* | *DNG701* |  | forward: 5-CACTGATTTCAACAGGGACA  reverse: 5’-AATAAGATACACCAAAGCC |
| *AGO1-1* | *AGO711* |  | forward: 5-TGGGAATGTTGCTGTAAGG  reverse: 5’-GGCGACTCAAACCGTCAGAC |
| *AGO1-2* | *AGO708* |  | forward: 5-GACCAGCCAAGCCATTCA  reverse: 5’-TCAAACATAACAGCACGATT |
| *AGO4-1* | *AGO705* |  | forward: 5-CTGATCCCATGTTGAGGGC  reverse: 5’-TTGAAGTTCCACCGACCA |
| *AGO4-2* | *AGO703* |  | forward: 5-TCCAAGGTCCCGACAATA  reverse: 5’-TCAACTAATAACTCCCGAATG |

* Website for the Chromatin Database: http://www.chromdb.org/
